# Incredible Women: Legal Systems Abuse, Coercive Control, and the Credibility of Victim-Survivors

**DOI:** 10.1177/10778012231220370

**Published:** 2023-12-17

**Authors:** Ellen Reeves, Kate Fitz-Gibbon, Silke Meyer, Sandra Walklate

**Affiliations:** 1 Department of Sociology, Social Policy and Criminology, School of Law and Social Justice, 4591 University of Liverpool, Liverpool, UK; 2 School of Social Sciences, Monash University, Melbourne, Victoria, Australia; 3 School of Health Sciences and Social Work, 5723 Griffith University, Brisbane, Queensland, Australia

**Keywords:** coercive control, legal systems abuse, feminist legal theory, criminal legal system, civil protection orders

## Abstract

Research has long demonstrated that victim-survivors of intimate partner violence face barriers to being believed when they seek help via the legal system and are simultaneously at risk of their abuser weaponizing the legal system against them. This article draws on the experiences of 54 women victim-survivors of coercive control in Australia who had experienced legal systems abuse within criminal and civil protection order systems. Drawing on feminist legal theory, we highlight that the legal system continues to disbelieve women and validate abusers. These experiences hold implications for victim-survivor views on the merits and risks of criminalizing coercive control.

## Introduction

Feminist legal theory is premised upon the assumption that the law is gendered, designed to serve the interests of white, middle-class men and to oppress those sitting outside of these parameters—particularly women (Graycar, 1990; Hudson, 2006). Unsurprisingly, in no area of the law is this more pronounced than in relation to *gendered violence*—and in particular, intimate partner violence (IPV). Indeed, decades of feminist research have captured the inability of the legal system to view women victim-survivors of such crimes as “credible witnesses” to their own victimization (see, inter alia, Epstein & Goodman, 2019; Gribaldo, 2014; Scutt, 1992), instead providing ample opportunities for male perpetrators to avoid accountability (see, inter alia, Douglas, 2018; Elizabeth et al., 2012; Fitch & Easteal, 2017). The complex nature of IPV intersects with broader structures of gender inequality and systemic biases within the legal system, rendering engagement with the law fraught with experiences of skepticism, delegitimization and disbelief for victim-survivors.

It is well-established that women victim-survivors seeking help from the legal system for IPV face a myriad of barriers to being believed, and when they are believed, to having action taken against the perpetrator (Douglas, 2019; Gezinski, 2020; Meyer, 2011). These barriers are exacerbated when the victim-survivor is experiencing “coercive control”, an insidious and often invisible form of IPV, which is largely incompatible with the incident-based and physical violence-focused criminal law response to IPV (Stark, 2007, 2012). Coercive control manifests in a range of compounding behaviors employed by the perpetrator, with one such behavior being “legal systems abuse”—the deliberate weaponization of the law by a perpetrator against the victim-survivor (Douglas, 2018). A key example of this, and the focus of this article, are the perpetrators’ efforts to have victim-survivors identified as predominant aggressors (see also Nancarrow et al., 2020; Reeves, 2020; Wangmann, 2009). This tactic raises important questions not just about why victim-survivors are disbelieved when they seek protection, but why perpetrators *are* believed, especially within a system that is guided by policy which clearly recognizes IPV as a gendered crime.

In this article, we consider gendered credibility through an examination of the qualitative accounts of 54 women victim-survivors who reported having experienced legal systems abuse in the criminal legal system (CLS) and/or civil protection order (CPO) system. The data is drawn from a larger survey dataset on Australians’ experiences of coercive control and their views on the merits and risks associated with its criminalization. The survey was conducted at a pivotal time in Australia, when the criminalization of coercive control was being widely discussed in the media, following the high profile killing of Queensland woman Hannah Clarke and her three children by her estranged husband in March 2020 (see further Bentley, 2022) and the publication of *See what you made me do* (Hill, 2021), both of which put the complexities surrounding women's experiences of domestic and family violence on the public agenda in unprecedented ways. At the time of data collection, Tasmania was the only Australian state or territory to have criminalized some coercive and controlling behaviors, specifically emotional and economic abuse (see McMahon & McGorrery, 2017). Clarke's death renewed calls for other Australian jurisdictions to enact laws similar to those recently introduced in England and Wales, Scotland and the Republic of Ireland (on evolving criminal law responses to coercive control, see McMahon & McGorrery, 2020; Stark & Hester, 2019; Wiener, 2022; on the approach taken to criminalizing coercive control across Europe, see EIGE, 2022, p. 45).

In this article, we examine women victim-survivors’ experiences of legal systems abuse within CLS and CPO systems. The lack of barriers that the women's abusive partners faced in having them identified as perpetrators and obtaining legal protection for themselves are contrasted with the barriers that the women themselves faced in being believed. The findings suggest that male perpetrators may, for a range of reasons, be perceived as more credible when they report IPV. That is not to say that men broadly are more credible when they report IPV victimization. There is an emerging body of research that documents the challenges male victim-survivors face in having the legal system take them seriously (Hine et al., 2020; Walklate et al., 2022). Instead, we argue that male *perpetrators* of coercive control, who are already skilled and adept in manipulation tactics, may be more readily believed when they make false claims to CLS/CPO actors, especially given that the woman's own reports may be fraught with the complex presentations of trauma which often serve to undermine her credibility (Gribaldo, 2014). Further, given that legal systems abuse is a commonly cited risk associated with the criminalization of coercive control (see, e.g., Fitz-Gibbon et al., 2023), we examine victim-survivors’ views on the merits of criminalization. This reveals that most, while in support of criminalization, hold significant concerns that the creation of a stand-alone offense will exacerbate legal systems abuse.

This article presents a valuable contribution to existing research on women's perceived credibility within the context of IPV victimization and offers a critical interrogation of the ways in which some male perpetrators turn to the law to further their control over victim-survivors as reported here in our sample. Importantly, it emphasizes the reality that simply changing the law to allow it to respond to more complex forms of IPV such as coercive control, does not necessarily mitigate the credibility deficits that women victim-survivors face. Efforts to criminalize coercive control across Australian states and territories need to be underpinned by a broad and in-depth understanding on behalf of CLS/CPO actors and service providers of the gendered nature of coercive control, and the ways in which the law and those operating within the system have the potential to replicate such dynamics.

## Coercive Control, Legal Systems Abuse, and Incredible Women: Examining Recent Research

Coercive control describes a form of IPV whereby: “…some combination of physical and sexual violence, intimidation, degradation, isolation, control and arbitrary violations of liberty are used to subjugate a partner and deprive her of her basic rights and resources” (Buzawa et al., 2017, p. 105).

The term was popularized by Evan Stark in 2007 and, while contested, draws on several decades of feminist research on men's violence against women in the home, centering themes of male privilege, power, and control (see also Dobash & Dobash, 1979; Ehrensaft et al., 1999; Schechter, 1982). Fundamental to the perpetration of coercive control is that the abuse is experienced as a *pattern*, rather than isolated incidents of abuse. Coercive control is widely understood to be an issue predominantly affecting women in their intimate relationships with men (Johnson, 2008; Myhill, 2015; Stark, 2007), however, emerging evidence demonstrates the perpetration of coercive control in other relationships contexts (Donovan & Barnes, 2020; Frankland & Brown, 2013; Reeves et al., 2023).

As part of the broader shift toward expanding the criminalization of IPV behaviors, some jurisdictions have criminalized coercive control (e.g., England and Wales, the Republic of Ireland, Scotland, on European approaches see EIGE, 2022, p. 45), while others have introduced standalone offenses of specific behaviors commonly observed in the context of coercive control (e.g., economic abuse and emotional abuse in Tasmania, Australia; see, inter alia, Wiener, 2022). This shift has been posited as a critical step in better responding to IPV and rests on the argument that broader legal responses are too focused on single incidents of physical violence and therefore limited in their ability to protect victim-survivors from nonphysical and ongoing forms of abuse (for discussion see Barlow & Walklate, 2022). Many argue that criminalization addresses such shortcomings, encouraging police officers to look at broader patterns of abuse and empowering prosecutors to pursue charges against perpetrators, particularly in cases where acts of physical violence are absent (for discussion, see Tolmie, 2018). This shift has been hotly debated amongst advocates, researchers, and victim-survivors, with some instead arguing that the nature of the law, evidentiary barriers, the ongoing shortcomings of police responses, and the potential for abusive perpetrators to manipulate such laws will render coercive control offenses ineffective and potentially harmful (Burman & Brooks-Hay, 2018; Walklate et al., 2018; Walklate & Fitz-Gibbon, 2020).

### Legal Systems Abuse as a Form of Coercive Control

A key concern surrounding the criminalization of coercive control is that the law will be used as a weapon by perpetrators who will seek to have the victim-survivor criminalized under the offense (Tolmie, 2018; Walklate et al., 2018; Watego et al., 2021). This concern stems, in part, from the potential of “legal systems abuse” as a control tactic used by perpetrators, specifically in the post-separation context (Douglas, 2018; Elizabeth et al., 2012; Fitch & Easteal, 2017; Miller & Smolter, 2011; Nancarrow et al., 2020; Reeves, 2020). Legal systems abuse:

…incorporates acts that are routinely used by batterers against their former partners to continue victimization and includes a range of behaviors such as filing frivolous lawsuits, making false reports of child abuse, and taking other legal actions as a means of exerting power, forcing contact, and financially burdening their ex-partners. (Miller & Smolter, 2011, pp. 637–638)

Douglas (2018, p. 85) highlights that this form of abuse often occurs in a separation and post-separation context, wherein victim-survivors’ efforts to use the law to navigate separation are weaponized against them:

The loss of opportunities for abuse that existed prior to separation and the engagement in litigation that co-occur around the point of separation creates a kind of perfect storm. While litigation may be an important part of women's search for safety and relationship closure, litigation can provide a new opportunity for perpetrators to continue to perpetrate abuse in a way that is legally justified.

Legal systems abuse has most frequently been documented in the family law system, particularly within the context of child custody proceedings, where gendered assumptions around mothering, fathering, and IPV provide fertile ground for male perpetrators’ abusive and controlling behaviors to flourish (see, inter alia, Elizabeth et al., 2012; Laing, 2017). Such behaviors serve to prolong the court process and ultimately intimidate and wear down the victim-survivor. This can result in the victim-survivor agreeing to orders which some evidence suggests are not necessarily in their best interests or that of their children (Laing, 2017). In the context of family law specifically, proceedings can continue for years—sometimes only ceasing when all children enter adulthood (House of Representatives Standing Committee on Social and Legal Policy, 2017). Legal systems abuse can lead to (or exacerbate) psychological distress, anxiety and depression, economic instability and poverty, and a loss of faith in the legal system (Douglas, 2018; Fitch & Easteal, 2017). As highlighted above by Douglas (2018), legal systems abuse is unique in that it can easily be interpreted as an abuser exercising his legal rights. This presents barriers for the law in recognizing and sanctioning the ongoing abusive behavior of the perpetrator (Fitch & Easteal, 2017).

### The Identification of Victim-Survivors as “Predominant Aggressors”

While much-existing literature on legal systems abuse highlights its occurrence within a family law context, such abusive tactics are not confined to the family law system. Perpetrators do utilize other areas of the law to control their partners, including the CLS and CPO systems. Research suggests that perpetrators of IPV may use the legal system to have their partner criminalized as a perpetrator (Gezinski, 2020; Goodmark, 2008; Mansour, 2014; Miller, 2005; Nancarrow et al., 2020; Reeves, 2020; Wangmann, 2009). They may do so by manipulating responding police officers into believing that *they* are the victim, engaging in behaviors such as ensuring that they are the first party to call the police, inflicting wounds onto themselves (or framing defensive injuries as offensive injuries), and/or painting the victim-survivor as mentally unwell (Miller, 2005; Nancarrow et al., 2020; Reeves, 2020). Such tactics often intersect with both personal and systemic biases held by the police, whose views and assumptions about IPV and women—particularly those from multiply marginalized backgrounds—may influence them finding the perpetrator more credible than the genuine or predominant victim-survivor (Gezinski, 2020; Nancarrow et al., 2020). Here, police may fail to take important steps to appropriately determine who the predominant aggressor is, such as conducting risk assessments, interviewing parties separately, and providing interpreters to victim-survivors who face language barriers (Reeves, 2020). It is important to note that perpetrators may also seek private CPO applications (e.g., without police support) against a victim-survivor as part of the abuse (Wangmann, 2009).

Studies show that socially disadvantaged and marginalized women, such as First Nations women, migrant and refugee women, women with disability, and women from low socioeconomic backgrounds may be most at risk of misidentification (Family Violence Reform Implementation Monitor, 2021; Mansour, 2014), with such findings being explained by the intersecting forms of discrimination faced by multiply marginalized women (Reeves, 2020). Stereotypes about some women held by responding police officers, who may also be ill-equipped to adopt context-driven and trauma-informed responses, assist in explaining why perpetrators are successful in employing these tactics.

Misidentification in criminal and CPO systems also impacts on ongoing family law proceedings (e.g., childcare arrangements and access matters), access to employment and education, access to housing, visa status, and economic stability (Nancarrow et al., 2020; Reeves, 2021). Additionally, the stigma associated with being labeled a perpetrator of IPV can take an emotional toll on victim-survivors, with some victim-survivors citing feelings of shame, embarrassment, exacerbated trauma, and a loss of faith in the system (Reeves, 2021).

### Incredible Women

To be a woman, whether as a victim of crime or accused, is to be incredible in the context of the law… (Scutt, 1992, p. 441)

Feminist legal theory reminds us that the law is gendered, designed to validate and serve the interests of the privileged men who make it over the women and minority populations who are often subject to it or have to use it for protection. Fundamental to this gendering of the law is the concept of credibility. Hudson (2006, p. 31) suggests that the law is not designed to protect persons “for harms that they suffer in virtue of their gender and/or race/ethnicity.” The male-ness of the law has meant that persons who experience crimes that sit outside of the white male middle-class experience, such as IPV, may face challenges in having their experience legitimized (Naffine, 1990; Renzetti, 1994; Scutt, 1992).

Epstein and Goodman (2019) employ narrative theory to highlight how the law and IPV are incompatible. Women victim-survivors may face challenges in meeting the criteria of “internal consistency” (e.g., the story is linear and logical) and “external consistency” (e.g., the story is consistent with the listener's experiences in the world; see also, Fitzgerald & Douglas, 2019; Fricker, 2007; Goodmark, 2008). In regard to the former, if a story is to be linear and logical to be credible, victim-survivors’ detachment, numbness, hyperarousal, and/or memory loss—all common trauma presentations (and in some cases symptoms of brain injury caused by the abuse)—may render them incredible when they tell the legal system what happened to them (Epstein & Goodman, 2019; Gribaldo, 2014). The latter, external consistency, draws on the idea that people “tend to assume that our personal experiences are universal: what we would likely do, say, and feel is what others would do, say, and feel” (Epstein & Goodman, 2019, p. 412). This presents a problem for women victim-survivors, who may come up against legal actors who perhaps due to their own gendered experiences of the world, cannot relate to experiences of IPV and further, cannot understand the barriers that women face in leaving an abusive relationship. This is encapsulated in Epstein and Goodman's (2019, p. 414) study wherein a magistrate presiding over a CPO case told a victim-survivor that “since I would not let it [IPV] happen to me, I can’t believe that it happened to you.”

Further, Goodman and Epstein (2019) consider the role of the victim-survivors *demeanor*, *motive*, and their *social location* in rendering their experience “believable.” Their analysis suggests that the credible victim-survivor finds the perfect balance between being emotional but not *too* emotional (demeanor), engages with the law to seek protection from the abuser but nothing else (e.g., financial compensation, custody of children) (motive), and holds high social standing—in that, negative (and often highly racialized and classist) stereotypes such as that of the “welfare queen” or the “gold-digger” are not applicable to her and therefore cannot lead to suspicion of her trustworthiness on behalf of the listener (social location).

By their gender alone women victim-survivors face a credibility deficit. However, intersectionality theory teaches us that this credibility deficit is exacerbated and compounded by some women's experiences and realities of race, class, migration status, disability, and sexuality, whereby these too differ from the dominant group's experience (Collins, 2019; Richie, 2012). The women who sit at these intersections of marginalization are both the most at risk of experiencing IPV (Australian Institute of Health and Welfare, 2019) and the most at risk of being disbelieved when they tell someone about it (Goodmark, 2008; Mansour, 2014; Nancarrow et al., 2020).

## Research Design

The data presented in this article draws on a larger survey of Australians’ (18 years and older) experiences of coercive control in a domestic and family violence context, and their views on the criminalization of coercive control (see also Reeves et al., 2021). The survey employed a mix of quantitative and qualitative measures examining participants’ experiences of abuse, help-seeking behaviors, and views on criminalization. The survey received 1,261 responses in total. The survey was advertised widely on social media and by IPV organizations in Australia, and open for a four-week period in May–June 2021. The results are not representative nor generalizable to the general population but do represent one of the largest research samples with victim-survivors of coercive control. This project received ethics approval from the Monash University Human Research Ethics Committee (project no. 27305).

### Participants

For the purposes of this article, which is focused on women's experiences of legal systems abuse by a male partner, the 1,024 responses from women victim-survivors constituted the first stage of data analysis. These entries were individually analyzed for themes of legal systems abuse in the CLS and/or CPO system. The decision to consider criminal law responses alongside CPO system response was informed by the Australian IPV policy and legal landscape. In Australia, the majority of CPO applications are initiated by the police, who are also tasked with enforcing breaches, which are criminal offenses (Crime Statistics Agency, 2023). It is also not uncommon for the police to apply for a CPO against a perpetrator concurrent to criminal charges and these matters will often be heard together in court (Crime Statistics Agency, 2023). Given the ways in which these systems overlap, and the key role of the police in both, we therefore have considered legal systems abuse within both systems in this article.

Participant responses were only included where they described having *directly* experienced being labeled a perpetrator within the CLS/CPO system. Participants who raised concerns about legal systems abuse as an unintended consequence of criminalization but had not experienced it themselves were excluded. A number of participants who cited their partner threatening to report them to the police but where this had not occurred were also excluded. The final sample included the experiences of 54 women victim-survivors. It is important to note that the survey did not specifically ask participants about experiences of legal systems abuse, and we anticipate that there were more women who had experienced this form of abuse but did not include it in their survey response (Table 1).

**Table 1.** Participant Demographics

Demographic	Percentage (number)
Age	
18–24	0 (*n* = 0)
25–30	1.85 (*n* = 1)
31–40	18.52 (*n* = 10)
41–50	40.74 (*n* = 22)
51–60	31.48 (*n* = 17)
61+	7.41 (*n* = 4)
Sexual identity^a^	
Heterosexual	88.8 (*n* = 48)
Gay, Asexual, and/or Questioning	0 (*n* = 0)
Lesbian	1.85 (*n* = 1)
Bisexual	9.25 (*n* = 5)
Queer	1.85 (*n* = 1)
Prefer not to say	1.85 (*n* = 1)
Australian State/Territory	
Victoria	40.74 (*n* = 22)
Tasmania	1.85 (*n* = 1)
Australian Capital Territory	1.85 (*n* = 1)
Western Australia	7.41 (*n* = 4)
New South Wales	20.37 (*n* = 11)
Queensland	20.37 (*n* = 11)
South Australia	5.56 (*n* = 3)
Northern Territory	1.85 (*n* = 1)
Type of area lived in	
Metropolitan	62.96 (*n* = 34)
Rural	9.26 (*n* = 5)
Regional	27.78 (*n* = 15)
Education	
Year 12 equivalent or less	18.52 (*n* = 10)
TAFE degree or undergraduate	38.89 (*n* = 21)
Postgraduate degree	40.74 (*n* = 22)
Prefer not to say	1.85 (*n* = 1)
Employment	
Full-time	40.74 (*n* = 22)
Part-time	18.52 (*n* = 10)
Casual	3.7 (*n* = 2)
Unemployed	18.52 (*n* = 10)
Student	1.85 (*n* = 1)
Other	14.81 (*n* = 8)
Prefer not to say	1.85 (*n* = 1)
Disability	
Has a disability	18.52 (*n* = 10)
Does not have a disability	77.78 (*n* = 42)
Prefer not to say	3.7 (*n* = 2)
Children	
Has children	94.44 (*n* = 51)
Does not have children	3.7 (*n* = 2)
Prefer not to say	1.85 (*n* = 1)

a Multiple responses allowed.

Participants experienced coercive control from a range of perpetrators, often multiple times. For all bar one participant, the perpetrator was a former partner, and it is these relationships that participants primarily focus on in their responses: 27.7% (*n* = 15) of participants also experienced coercive control from a parent, 11.1% (*n* = 6) from an in-law, 7.4% (*n* = 4) from a daughter, and equally 3.7% (*n* = 2) from a current partner, a sibling, a son and another family member. One participant (1.85%) experienced coercive control from a grandparent. For those participants who identified as lesbian, bisexual or queer (*n* = 6), all bar one referred to intimate relationships with male partners.

### Limitations

A limitation of this study is that the sample is relatively homogenous which is reflective of the larger survey sample for this study (Reeves et al., 2021). While the homogeny of the sample, which largely speaks to the experiences of white, heterosexual, highly educated women living in metropolitan areas, tells us that coercive control and legal systems abuse transcends social location, we pose it here as a limitation given that extensive research shows that multiply marginalized women, such as women of color, women with disability, migrant and refugee women and women from low socioeconomic backgrounds are at an increased risk of being criminalized as perpetrators (see, inter alia, Goodmark, 2023; Tolmie et al., 2018). We are conscious of the fact that some groups are under-represented in our sample.

### Data Analysis

The results presented in this article draw on the qualitative data captured in the survey. Nvivo qualitative data analysis software was used to conduct thematic coding. Given that no specific questions were asked about experiences of legal systems abuse, entire survey responses were coded. However, participant experiences of legal systems abuse tended to be discussed under open-ended questions exploring legal help-seeking. The initial coding framework was broad, speaking to the utilization of grounded theory (Glaser & Strauss, 1967)—we created themes as they presented themselves in the data. However, with the theme of credibility at the fore, secondary analysis narrowed the focus to two dominant themes: women's experiences of abusers’ perpetration of legal systems abuse; and views on the criminalization of coercive control in regards to legal systems abuse. These key themes are each discussed in the presentation of the research findings below.

## Findings

### Experiences of Legal Systems Abuse

In many instances, as described by the women victim-survivors in this sample, the abuser was successful in shaping themselves as the predominant victim in the relationship, whereas the women were not believed. This is captured in the following reflections of two victim-survivors:

They didn’t believe me. They took me away in their police car and locked me up in a psychiatric hospital because my abuser told them I was mentally ill and delusional. Other times I reported the police made fun of me or blamed me for the abuse. (Participant 23)

They did nothing and then charged me when he lied and said I had assaulted him. All going to the police did was empower him to think he could get away with anything, which he did. (Participant 36)

These quotes highlight perpetrators’ strategic manipulation of legal responses as a further mechanism to abuse, disempower, and at times silence the victim-survivor.

In the above quote from Participant 36, the abuser was a serving police officer. Participant 32 and Participant 35 also experienced coercive control from a police officer, and they felt that this contributed to their criminalization:

Initially they gave me a [CPO] but the day before the hearing they struck an agreement with my ex. He is also a serving police officer. The police agreed both parties would sign undertakings. I refused to comply as this was done with no consent from me. The case was dropped. (Participant 32)

My ex is a policeman … [H]is friends served me with [CPOs]. [I]t has been biased in his favour from day one. (Participant 35)

Participants whose abuser was a serving police officer spoke about how easy it appeared to be for their abuser to manipulate responding police officers. However, such experiences were not limited to (ex)partners of police officers. Participant 45 was physically assaulted by her former partner during child handover. When she returned home, she called the police, but they had already attended her former partner's house because a neighbor had called them during the assault. She explained:

I asked them to charge him with assaulting me and they said they would not because “he was crying”. I said that's because he was on his final warning from me because he's assaulted me so many times - he was crying because he thought you were going to arrest him. I was stressed out, scared that he was going to come around and kick the door down, and I have these young coppers telling me how they’re not going to charge him because he's “crying”! (Participant 45)

A more senior police officer later apologized to this participant and she eventually obtained an CPO, however, her former partner was then successful in obtaining a retaliatory order which “helped him build a narrative that he was not to blame for any of his behavior, that he was just under a lot of stress” (Participant 45).

Participants frequently suggested that when their perpetrators obtained a CPO and/or criminal charges against them in response to the victim-survivor's help-seeking attempts, the perpetrators listed behaviors that they had actually subjected the victim-survivor to. As two victim-survivors recounted:

He took a counter order against me, accusing me of the things he had done to me - you see, they know exactly what they are doing and how to manipulate the system. There was no help, no recourse. (Participant 47)

Even this [CPO] he put on me, the words he's put in there are the words he's said to me over the 24 years. (Participant 5)

Participant 9 said that she frequently had the police called on her by her former partner, despite the fact that she had had a number of conversations with police about her safety and she had done “everything they asked of me.” She spoke of a double standard in the police response:

If the children behaved badly on this time, he took the children to the [police] station to file a complaint against me. If I asked them [the police] to stop doing that … they shrugged their shoulders. If my children are not delivered as per court orders, they [the police] won’t help me. It's a bizarre double-standard. And I’m polite. I’m kind. I’m gentle. I would never hurt a soul. I never have. Yet I can’t get help. And I’m arrested. It's the most insane reverse logic. It's as though the victim has to have a degree in law, policing, psychology and abuse. Yet the abuser just remains a brainless thug. (Participant 9)

This double standard was reflected in the experiences of other victim-survivors in the study, such as Participant 4, who described:

Police were abhorrent. They allowed him to manipulate them into dealing with me regularly. When I complained I was told they couldn’t do anything. But he seemed to be able to make them do anything.

It is important to note that a number of participants stated that perpetrators were ultimately unsuccessful in their efforts to criminalize/obtain a CPO against them, with the cases being dismissed in court. For example, Participant 8, who became subject to a CPO application after pursuing fraud charges against her abuser, stated:

He provided no evidence and they [police] believed him and he was given an application for [a CPO] … I then at a cost of $12000 [AUD] had to defend myself in court. The [CPO] application was thrown out of court as soon as the magistrate heard from him on the stand that he had contacted the police following being contacted about the fraud. However, he was believed over me and the police stopped investigating the fraud and instead focussed on me.

In this instance, while the perpetrator was not able to obtain an [CPO], it is evident how his claims of victimization ultimately undermined the participant's safety, and her own charges against him for fraud were withdrawn. The fact that perpetrators may not be successful in court suggests that magistrates may be becoming more alert to the perpetration of legal systems abuse by male abusers. However, it can also be inferred that CPO applications protecting perpetrators are commonly being initiated (either by the police or privately) where there is minimal evidence to support it. Women victim-survivors, in contrast, faced significant barriers in being believed when they pursued the same legal avenues for their protection.

Many participants who experienced legal systems abuse described a loss of faith in the CLS/CPO systems. This was often tied to the sense that the account of their perpetrator was more readily believed over their own. For example, Participant 44 spoke about a loss of faith in the police:

I will never seek the assistance of police for anything. They do more harm than good and rarely do anything but protect, enable and continue the abuse. The police made everything worse for me mentally and emotionally. They verbally abused me and threatened to charge me with assault for a scratch on his neck from trying to get him to stop choking me.

Participant 8 spoke about the lasting impacts of misidentification, citing an experience 18 months after legal system involvement where she called the police because someone was trespassing on her property (a non-IPV related incident), but the police did not attend. She explained:

I believe because they continue to believe what my ex told them and is recorded on the … system against my name. Who knew you could say what you like about another person to the police and it gets recorded against their name and is then used to determine how they are treated from that point onwards. Well you can do this if you are a male. When I presented solid evidence of criminal action no action was taken. I no longer rely on the police and am frightened of them and regard them as abusive. (Participant 8)

The above quote highlights the lasting effects of legal systems abuse on victim-survivors subsequent help-seeking attempts, deteriorating victim-survivors’ faith in the legal system. This lack of faith is consistent with larger research on victim-survivors’ experiences with the legal system, however, such feelings are likely to be exacerbated for victim-survivors who are criminalized as perpetrators.

### Views on the Criminalization of Coercive Control with Regards to Legal Systems Abuse

While the majority of victim-survivors in this sample stated that they supported the criminalization of coercive control (88.46%, for further analysis of this data see Reeves et al., 2021), when the qualitative comments are interrogated, the study reveals victim-survivors’ significant reservations for the potential legislation and its effectiveness, particularly in relation to its ability to ameliorate the risks of legal systems abuse.

#### Criminalization as a Tool to Address Legal Systems Abuse

For those victim-survivors who supported the introduction of a new coercive control criminal offense, a number reflected that had coercive control been criminalized when they were seeking help, they would not have been misidentified or have experienced legal systems abuse. As two participants described:

The abuser would have been identified as an abuser and not had the ability to convince the system to the contrary. The abuser would not have been awarded custody of our 2 young children to withhold and alienate from their mother. The abuser would not be currently raising 2 more humans to think this is acceptable behaviour. […] Ironically, he is still using similar forms of coercive control on court participants as used on myself and our children. And it still goes unrecognised. (Participant 20)

I wouldn’t have had the abuse by proxy via the legal system. (Participant 37)

Broadly, some participants suggested that the criminalization of coercive control would provide fewer opportunities for perpetrators to commit systems abuse:

Awareness and laws in place would make a huge difference I think and prevent the perpetrator using the system to enable his abuse. (Participant 14)

It will reduce the misidentification and criminalisation of victims. It will help provide better protection and outcomes in family court. (Participant 54)

These findings highlight victim-survivors’ expectations that criminalization will increase legal practitioner, judicial officers and law enforcement awareness and understanding of coercive control and in return minimize opportunities for perpetrators to abuse the legal system as another tool of IPV. Victim-survivors further believed that such a shift would lead to being believed more readily by actors in the CLS/CPO system. For example, Participant 27 stated:

I would have had more confidence that I would be listened to and heard AND that something could actually be done about it.

It may be that as participants in this study had experienced coercive control, and coercive control laws are designed to recognize and respond to this specific form of abuse, they believe that the law will act as it is intended and provide greater opportunities for victim-survivors of coercive control to be believed. Following this logic, if *they* are believed, their abusive partners are less likely to be. Further, it may be that participants believe that legal systems abuse will fall into the purview of coercive control legislation, or at least understood within the context of coercive control. While these are extrapolations from the study data, we note the importance of understanding victim-survivors’ expectations of the impact of the law *in action*, if the goals and objectives of any law-making process are to be achieved.

#### Concerns About the Criminalization of Coercive Control With Regard to Legal Systems Abuse

As noted above, a significant majority of this subsample supported the criminalization of coercive control in principle, however, the qualitative responses to questions around criminalization paint a different picture. Of specific relevance to this article, there was a greater tendency for participants to express concerns about criminalization than there was for participants to show support without also raising concerns. Perhaps unsurprisingly given the nature of this subsample who had all experienced legal systems abuse, these concerns were primarily based around the exacerbation of the issue of misidentification. For example, a number of participants stated that they would not pursue charges under a coercive control offense due to fears of perpetrator manipulation of the law:

I still would have been [too] scared. He may have turned it around and lied and I would be the person in trouble. Offenders are gifted liars. (Participant 17)

In my case I saw how easily someone who has a personality disorder that makes them use [coercive] control can turn this on the victim. Just as I was the one who ended up in court [defending] myself against [a CPO] because he lied to the police he could just as easily have used [coercive] control laws in the same way. People who really have never experienced someone like this expect[s] them to be honest and tell the truth, or to show signs of lying and do not realise they can calmly lie about anything to manipulate someone with no conscience. If there were laws I would have been the one jailed, just as my name with the police contains a number of untrue entries with false information (and I cannot get these changed). (Participant 8)

Some participants spoke less about their direct experience, and more broadly about the potential unintended consequences of criminalization. As captured in the responses provided by two victim-survivors:

It will give coercive control perpetrators another avenue of coercive control - having the victim charged. (Participant 36)

I think the laws need to be very carefully considered, and the enforcement of them needs to be rigorous. I can foresee a situation where perpetrators use the Coercive Control laws to portray themselves as the victims. (Participant 42)

For a number of participants, their loss of faith in the police and the legal system shaped their skepticism of a new coercive control offense. As two victim-survivors described:

It sounds good in theory but new laws won’t work because the people in power won’t follow or enforce them. If they do, it will be used against victims. (Participant 23)

Misogynistic police/court culture needs to be fixed, and police accountability. High levels of victims are charged already and that could worsen. Need to be careful. (Participant 36)

The views around criminalization illustrate victim-survivors’ grave concerns that the systematic gaps, currently allowing for coercively controlling perpetrators to manipulate and abuse the legal system without the existence of a criminal offense of coercive control, may facilitate even greater rates of misidentification once such an offense is in place. Hence highly skilled and manipulative perpetrators would have another tool at their disposal for legal systems abuse.

Findings addressing support for, as well as concerns around the criminalization of coercive control, are largely consistent with the perspectives of the broader survey sample. However, for victim-survivors in this subsample their views were often tied to, and directly informed by, their experiences of misidentification and legal systems abuse.

## Discussion

This article highlights women victim-survivors’ experiences of legal systems abuse in CLS and CPO systems. In contrast to women's challenges in being believed for their own victimization experiences, which is widely documented in existing literature (see also Douglas, 2019; Gezinski, 2020; Meyer, 2011), women felt that the same barriers were easily circumvented by their perpetrator. Narrative theory, employed in the context of IPV, suggests that lesser-known experiences of IPV, such as coercive control, may impede on the listener's ability to relate to women's experiences (Epstein & Goodman, 2019). However, the fact that perpetrators were able to have their allegations validated suggests credibility is gendered, wherein the listener (often police), may view men's experiences of coercive control as more “consistent” than women's. This may be due to the perpetrator's skill in manipulation techniques, leading to more linear and logical explanations—contrasted to women's accounts that present a less “consistent” narrative in the aftermath of abuse and related trauma (Epstein & Goodman, 2019). Fricker's (2007) concept of identity power is useful in understanding the findings presented in this article. Fricker (2007, pp. 16–17) argues that:

… identity power is an integral part of the mechanism of testimonial exchange, because of the need for hearers to use social stereotypes as heuristics in their spontaneous assessments of their interlocutor's credibility.

While identity power underpins men's ability to control women in intimate relationships (and beyond), it also impacts on women's ability to be treated as credible witnesses to the control that men have exercised over them when they seek help through the legal system. It is well established that legal system actors sometimes draw on and are influenced by stereotypes about women when responding to IPV—for example, that women are overly emotional, illogical, and erratic, and when they raise allegations of abuse, that they do so vindictively (Epstein & Goodman, 2019; Gezinski, 2020; Gribaldo, 2014; Hartman & Belknap, 2003; Melville & Hunter, 2001). For multiple marginalized women, these stereotypes are compounded (Durfee, 2020; Nancarrow, 2019; Richie, 2012). By virtue of identity power, the abusers of the women in this study may not have encountered these same stereotypes. Just as they were able to coercively control their partners, they were also emboldened by the system to commit legal systems abuse, with their false allegations and accounts of abuse as reported by our participants facing less scrutiny. This suggests that despite laws being put in place to protect women, the continued gendered operation of the law impedes on its ability to treat women victim-survivors as genuine witnesses to the violence committed against them.

This raises questions about the implications of a coercive control offense. Indeed, while a number of victim-survivors in this study showed support for criminalization, believing that such a law would enhance their credibility and give the legal system the tools to believe them and take action against the genuine perpetrator, the majority of participants had doubts as to whether a coercive control offense would minimize the perpetration of legal systems abuse. For many, a lack of faith in the police to accurately identify the predominant aggressor and the person most in need of protection, informed through their lived experience of misidentification, shaped their view that a coercive control offense could offer perpetrators another vehicle through which to exacerbate misidentification and the criminalization of victim-survivors.

The findings from this study also capture the ways in which women's experiences of legal systems abuse shaped their help-seeking behaviors. Women spoke about how being labeled a perpetrator of IPV influenced their lack of trust in, and lack of willingness to reengage with the police not only for IPV matters, but also for non-IPV related matters. This highlights the ways in which this loss of faith is likely to be compounded for women who are not only disbelieved or dismissed when they report abuse, but also face criminalization for crimes that they have been victim to.

A number of participants in this study cited their abuser's legal systems abuse being intercepted in court, wherein magistrates ultimately decided that there was insufficient evidence to grant the abuser a CPO or proceed with criminal charges against the victim-survivor despite police having pursued these initially. This finding supports the contention that male abusers face less scrutiny at earlier stages of the CLS/CPO system in their efforts to criminalize victim-survivors. Thus CPO applications and criminal charges are being brought forth against victim-survivors by police despite minimal supporting evidence. The unsuccessful efforts of (some) perpetrators suggest that magistrates may be more attuned to legal systems abuse than police (see also, Nancarrow et al., 2020), or perhaps that legislative requirements (e.g., a CPO can only be made where IPV has been committed and is likely to be committed again in the future) place greater limitations on judicial officers than on the police. However, even where applications or charges are dismissed at court, the impacts of legal systems abuse are still felt by victim-survivors (see, Reeves, 2021). For this reason, among others, relying solely on the court to correct mistakes made by the police is not an effective way for the legal system to address this form of abuse.

## Conclusion

This article examines victim-survivors’ experiences of legal systems abuse, and their views on criminalization, in the context of ongoing debates concerning the criminalization of coercive control in Australia. To do so, the article draws upon the findings of a 2021 survey which while not asking victim-survivors directly about their experiences of misidentification or legal systems abuse, collected significant data on this specific topic. We present the views and experiences of 54 women victim-survivors who experienced legal systems abuse in the CLS/CPO system—specifically, women who had faced charges for IPV-related offenses and/or who were listed as a respondent on a CPO/CPO application. The results demonstrate the ways in which the perpetrator's weaponization of the legal system plays a key role in victims’ experiences of coercive control, especially in a post-separation context. Legal systems abuse inherently relies on the law believing the accounts of perpetrators (often men) over those of victim-survivors (often women). This article adds to this body of research on the incredibility of women in a legal context, especially when they have been subject to, and are seeking protection from, gendered violence (Epstein & Goodman, 2019; Scutt, 1992). The CLS/CPO system in Australia evidently faces an ongoing challenge of balancing the needs of genuine victim-survivors with perpetrators’ rights as citizens to engage with the legal system (Douglas, 2018) and carries implications for jurisdictions with similar systems across the globe. As this study documents, this challenge is exacerbated by the continued relegation of women victim-survivors to a position of incredibility and the failure of the legal system to locate IPV within gendered social structures.
